# The complete chloroplast genome sequence of *Dendrobium densiflorum* and its phylogenetic implications

**DOI:** 10.1080/23802359.2020.1816511

**Published:** 2020-09-04

**Authors:** Liu Wei, Niu Zhitao, Xue Qingyun, Zhang Benhou, Ding Xiaoyu

**Affiliations:** College of Life Sciences, Nanjing Normal University, Nanjing, China

**Keywords:** *Dendrobium densiflorum*, Australasian clade, chloroplast genome, phylogeny

## Abstract

*Dendrobium* is one of the largest genera in Orchidaceae with approximately 1500 species. Many of them have been used as Traditional Chinese Medicine (TCM) for hundreds of years. Here, the first complete chloroplast genome sequence of *D. densiflorum* was reported and characterized. The complete cpDNA of *D. densiflorum* is a circular molecule of 153,122 bp, which contains 76 protein-coding genes, 30 tRNA genes, and four rRNA genes. Our phylogenetic analysis indicated that the newly sequenced cpDNA of *D. densiflorum* could be used for the phylogenetic study of *Dendrobium* species.

Chloroplast genomes (cpDNAs) of seed plants, in general, have relatively small sizes, conserved gene contents, dense coding regions and slower evolutionary rates as compared to nuclear and mitochondrial genomes (Wolfe et al. 1987). These unique features mentioned above have led the cpDNA be a focus of research in plant molecular evolution and systematics (Jansen et al. 2007). *Dendrobium*, a genus belongs to the tribe Dendrobieae (Orchidaceae: Epidendroideae), is one of the largest genera in Orchidaceae with approximately 1500 species mainly distributed in tropical Asia, Australasia, and Australia. Because of their beautiful and long-lasting flowers, *Dendrobium* species are valuable commercially. Moreover, many species in this genus have been used as Traditional Chinese Medicine (TCM) for hundreds of years due to their excellent medicinal merits, i.e., *D. densiflorum*. Recently, the cpDNA sequences are available for more than 30 *Dendrobium* species; however, there still none information about the chloroplast genome sequence of *D. densiflorum*. (Luo et al. 2014). Therefore, to provide more insight into the cpDNA structure and evolution study of *D. densiflorum*, we sequenced the complete cpDNA sequence and reported the cpDNA features of *D. densiflorum*.

In this study, the total genomic DNA was extracted from 2 g fresh leaves of *D. densiflorum* (voucher number: LW20190816), and sequenced on Illumina Hiseq 4000 sequencer. The raw reads were trimmed and mapped to the reference cpDNA sequence of *D*. *officinale* (NC_024019) according to the method in Niu et al. (2017). The gaps and boundaries of IRs were confirmed by PCR assays. The complete cpDNA sequence has been submitted to DDBJ under the accession number of LC528135.

The complete cpDNA of *D. densiflorum* is a circular molecule of 152,110 bp. The GC content is 37.13%. It has a typical quartered structure with a pair of inverted repeats (IRs) (26,124 bp each) separated by a large single copy (LSC) (85,078 bp) and a small single copy (SSC) (14,785 bp) regions. The GC content of LSC, SSC and IR were 34.95%, 30.27% and 43.30%, respectively. A total of 111 unique genes were successfully annotated, including 76 protein-coding genes, 30 tRNA genes, and four rRNA genes. Among the eleven *ndh* genes, only *ndhB* genes in IR regions were functional with full reading frames, whereas others were truncated or completely lost.

We constructed the maximum-likelihood (ML) tree of the whole cpDNA sequences of 11 *Dendrobium* species using RAxML 8.0.2 (Stamatakis 2014) with *Phalaenopsis aphrodite* (NC_007499) and *Phalaenopsis equestris* (NC_017609) as outgroups. As shown in Figure 1, the ML tree has revealed a sister relationship between *D. densiflorum* and *D*. *exile*, which was consisting with previously reported results (Xiang et al. 2013), which indicated that the newly sequenced cpDNA of *D. densiflorum* could be used for the phylogenetic study of *Dendrobium* species.

**Figure 1.**
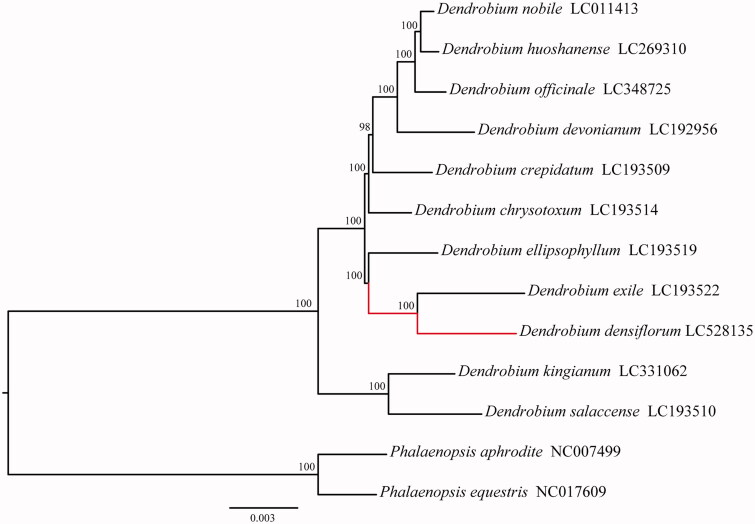
Maximum-likelihood tree of 11 *Dendrobium* species based on the whole cpDNA sequences.
